# The Multivariate Physical Activity Signatures Associated With Self-Regulation, Executive Function, and Early Academic Learning in 3–5-Year-Old Children

**DOI:** 10.3389/fpsyg.2022.842271

**Published:** 2022-04-05

**Authors:** Kristoffer Buene Vabø, Katrine Nyvoll Aadland, Steven James Howard, Eivind Aadland

**Affiliations:** ^1^Department of Sport, Food, and Natural Sciences, Faculty of Education, Arts, and Sports, Western Norway University of Applied Sciences, Bergen, Norway; ^2^Early Start and School of Education, University of Wollongong, Wollongong, NSW, Australia

**Keywords:** cognition, preschool (kindergarten), accelerometer, self-regulation, executive function, learning

## Abstract

The evidence regarding associations between intensity-specific physical activity and cognitive and learning outcomes in preschoolers is inconsistent and limited by low sample sizes and analytical approaches that cannot handle the multicollinearity among multiple physical activity intensity variables. We aimed to determine the multivariate physical activity intensity signatures associated with self-regulation, executive function, and early academic learning in preschool children aged 3–5 years. A 711 Norwegian preschool children (mean age 4.6 years, 52% boys) provided valid data on physical activity (ActiGraph GT3X+), self-regulation, executive function, and early academic learning during 2019–2020. Multivariate pattern analysis was used to determine associations between uniaxial and triaxial intensity spectra (time spent in intensities from 0–99 to ≥15,000 counts per minute) and the outcomes in the total sample and in subgroups split by sex and age (median split). Uniaxial data led to the highest explained variances (*R*^2^) and were reported as the primary findings. We found significant association patterns between physical activity and numeracy (*R*^2^ = 4.28%) and inhibition (*R*^2^ = 1.48%) in the total sample. The associations with numeracy were negative for time spent sedentary (0–99 counts per minute) and positive for time spent in moderate to vigorous intensities (≥ 1,000 counts per minute). The associations with inhibition were positive for time spent sedentary (0–99 counts per minute) and in vigorous intensities (≥ 8,500 counts per minute) and negative for time spent in low to moderate intensities (100–3,499 counts per minute). Associations with numeracy were stronger in boys (*R*^2^ = 5.58%) and older children (*R*^2^ = 7.27%), and associations with inhibition were stronger in girls (*R*^2^ = 3.12%) and older children (*R*^2^ = 3.33%). In conclusion, we found weak associations with numeracy and inhibition across the physical activity intensity spectrum in preschool children.

## Introduction

Cognitive function, defined as the set of mental processes that contribute to perception, memory, intellect, and action, is considered to be a key health indicator at early age (Khan and Hillman, 2014; Donnelly et al., 2016; Poitras et al., 2016; Carson et al., 2017) and an early marker for later life success (Moffitt et al., 2011). In recent years, research within psychology, neuroscience, and education has focused on how the processes of self-regulation and executive function relate to reasoning, problem solving, and goal-directed behaviors (Zelazo et al., 2016). Self-regulation is generally defined as the capability to control or direct one’s attention, thoughts, and emotions, despite competing impulses or distractions (McClelland and Cameron, 2012). Self-regulation depends on executive function, which is often understood as three related, but distinct fundamental components: inhibition, working memory, and cognitive flexibility (Miyake et al., 2000; Best, 2010; Diamond, 2013; Zelazo et al., 2016). Both self-regulation and executive function seem to impact the development of early academic learning skills such as vocabulary and initial mathematical concepts in young children (Zelazo et al., 2003; Blair and Razza, 2007; McClelland et al., 2007).

There is evidence of positive associations between physical activity (PA) and cognitive function in school-aged children and youth; however, evidence on associations for PA with academic achievement is inconsistent (Rasberry et al., 2011; Donnelly et al., 2016). In younger children, the evidence on associations for PA with cognition and learning is weaker and overall less consistent (Timmons et al., 2012; Carson et al., 2016, 2017; Wood et al., 2020; St. Laurent et al., 2021). Yet, the preschool years are crucial with respect to developing cognitive skills (Thompson and Nelson, 2001; Diamond, 2006; Calkins, 2007; Eisenberg and Zhou, 2016). As the brain is notably susceptible to stimuli in this period (Zelazo et al., 2016), there is a need to further investigate the relationship between PA and cognitive development in this age group. A recently published systematic review by St. Laurent et al. (2021) reports that few studies have explored cross-sectional associations between objectively determined PA and cognitive and academic outcomes in children younger than 6 years old. Relevant studies show a tendency for positive associations between PA and self-regulation (Becker et al., 2014; Bai et al., 2021), while associations between PA and executive function are mixed (McNeill et al., 2018; Willoughby et al., 2018; Cook et al., 2019; Bezerra et al., 2021), and associations between PA and early academic learning are positive (Bai et al., 2021) or non-existent (Becker et al., 2014; St. Laurent et al., 2018).

Previous studies exploring associations for PA with cognition and academic achievement in children vary methodologically and generally have low sample sizes (Howie and Pate, 2012; St. Laurent et al., 2021), which may lead to confusion and lack of generalizability of their results (Kim et al., 2012; Cain et al., 2013). PA as measured by accelerometry is usually reported as time spent along the intensity spectrum, including sedentary time (SED) and one or more PA intensities light-intensity PA (LPA), moderate-intensity PA (MPA), vigorous-intensity PA (VPA), and/or moderate to vigorous-intensity PA (MVPA). However, intensity cut points have only been validated for a certain combination of axes and epoch lengths (Migueles et al., 2017). Thus, there is a lack of agreement on the most appropriate cut points for preschool-aged children (Kim et al., 2012). Moreover, all studies using accelerometry in the systematic review by St. Laurent et al. (2021) used a 15-s epoch length. As children have a natural sporadic PA pattern characterized by intermittent bursts of PA generally lasting less than 10 s (Bailey et al., 1995; Vale et al., 2009), a summation of PA over longer periods will misclassify time spent in the lower and higher end of the PA intensity spectrum. A short epoch length (1 s) has been shown to better capture information about PA of relevance for cardiometabolic health than longer epoch lengths in schoolchildren (Aadland et al., 2020a). A 1-s epoch length has also been suitable to determine associations between the PA intensity spectrum and body mass index (BMI; Aadland et al., 2021) and fundamental motor skills (Nilsen et al., 2020) in preschool children.

Multiple linear regression is commonly used to determine associations between PA intensities and outcomes. However, since different PA intensity variables derived from accelerometry are strongly correlated, novel approaches are needed to model the joint contribution of multiple intensity variables. Multivariate pattern analysis can handle multiple multicollinear PA intensity variables and makes it possible to study associations for the uniaxial (i.e., vertical axis only) or the entire triaxial intensity spectrum with outcomes (Aadland et al., 2018, 2019b, 2021; Nilsen et al., 2020). The inclusion of full intensity spectra from triaxial accelerometry has been shown to be a more powerful approach than uniaxial accelerometry and traditional intensity variables to capture PA of relevance for cardiometabolic health and BMI in children (Aadland et al., 2019b, 2021). Multivariate pattern analysis has been used to determine association patterns between the PA spectrum and fundamental motor skills (Nilsen et al., 2020) and BMI (Aadland et al., 2021) in preschoolers but has not been applied to determine associations of the PA intensity spectrum with self-regulation, executive function, and early academic learning.

The aim of this study was to determine multivariate association patterns of both uniaxial and triaxial PA intensity spectra (including SED) with self-regulation, executive function, and early academic learning in a large sample of preschool-aged children. Based on the available literature, we hypothesized to find weak associations patterns with the outcomes across the PA intensity spectrum, with results showing favorable associations for moderate and vigorous intensities and unfavorable associations for SED.

## Materials and Methods

### Study Design, Recruitment, and Participants

**Figure 1:**
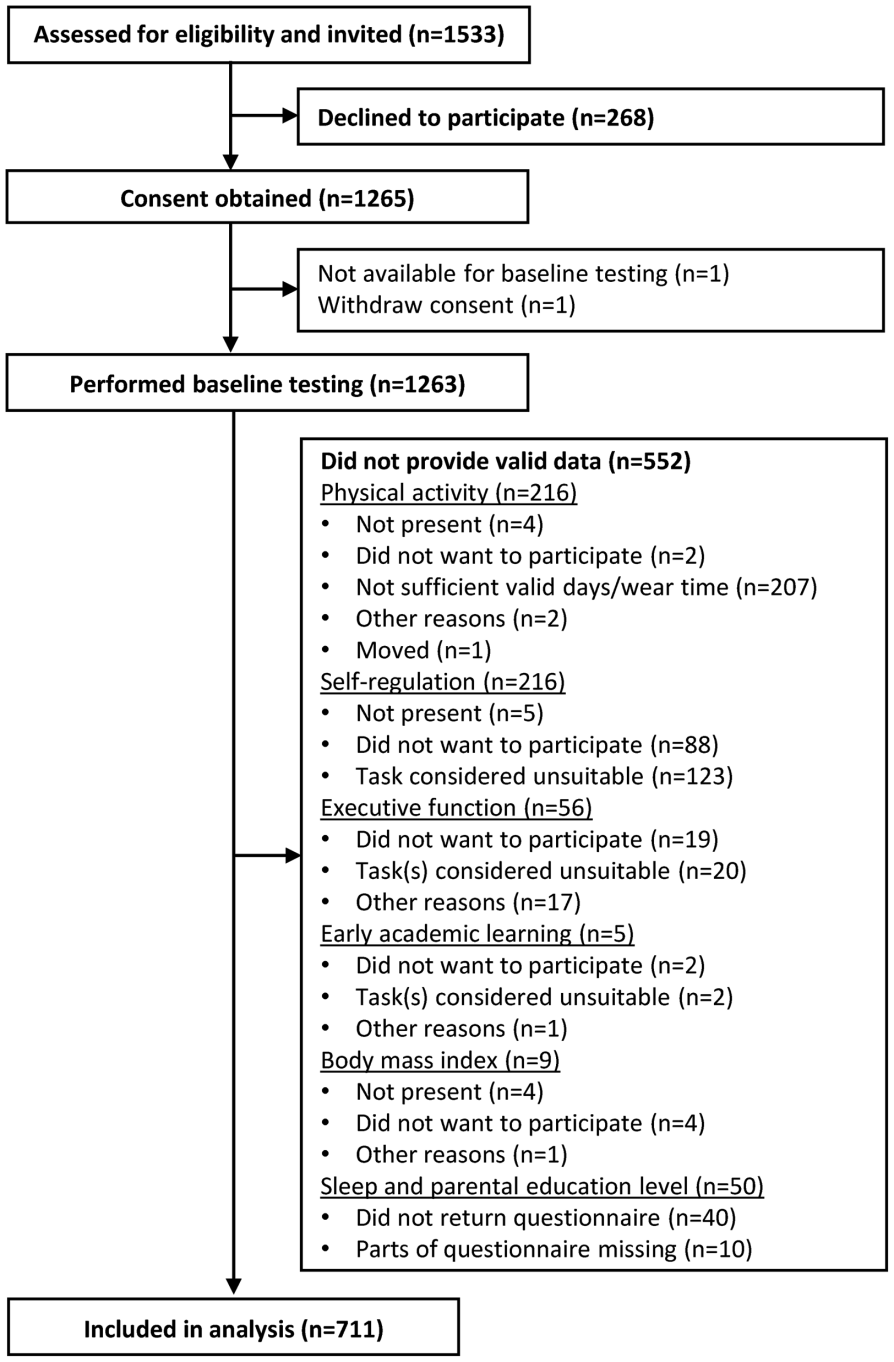
Flow chart of recruitment and the missing data (*n* = children). Other reasons include accelerometer not returned (PA), technical errors, automatically removed data (The Go/No-Go task), and unknown reasons.

The present study has a cross-sectional design and used baseline data obtained from preschoolers in the Active Learning Norwegian Preschool(er)s (ACTNOW) cluster randomized controlled trial. A total of 56 preschools were invited to participate, of which 46 preschools (82.1%), encompassing 1,532 children (3–5-year-olds), agreed to participate. At baseline, 1,263 (658 boys and 605 girls) children were recruited and agreed to participate (participation rate, 82.4%). Of these, 711 children (369 boys and 342 girls) provided valid data on all variables relevant to the present study. The main reasons for missing data were that children had insufficient accelerometry wear time, did not want to participate on one or more tasks, or the trained assessor considered the task(s) to be unsuitable for the children due to their young age, language barriers, and/or other mental/physical disabilities (Figure 1).

### Procedures

We have previously published a detailed description of the study protocol (Aadland et al., 2020c) and therefore provide only a brief overview of the relevant procedures herein.

#### Physical Activity and Sedentary Time

PA and SED were measured using ActiGraph GT3X+ accelerometers (ActiGraph, LLC, Pensacola, Florida, United States; John and Freedson, 2012). Children were instructed to wear the accelerometer on their right hip 24 h a day for seven consecutive days, included while sleeping, but except during water activities (i.e., swimming and showering). Accelerometers were initialized at a sampling rate of 30 Hz and analyzed at a 1-s epoch length using MATLAB. Consecutive periods of ≥ 20 min of zero counts were defined as non-wear time (Esliger et al., 2005). Children having ≥ 480 min/day of wear time accumulated between 06:00 and 22:00 for ≥ 3 weekdays and ≥ 1 weekend day were included in the analysis (Aadland et al., 2020b).

For the descriptive statistics and bivariate correlation analysis, we reported SED (≤ 100 cpm), total PA [TPA, counts per minute (cpm)], and time (min/day) spent in intensity-specific PA, LPA (101–2,295 cpm), MPA (2296–4,011 cpm), VPA (≥ 4,012 cpm), and MVPA (≥ 2,296 cpm), as proposed by Evenson et al. (2008) (vertical axis only). Further, to capture movement in narrow intensity intervals, we included the whole PA spectrum from all axes (vertical, antero-posterior, and medio-lateral) and created 33 PA variables of total time (min/day) for each axis; 0–99, 100–249, 250–499, 500–999, 1,000–1,499, 1,500–1,999, …, 14,500–14,999, and ≥ 15,000 cpm. For comparability with previous studies, we interpreted PA intensities along the intensity spectrum of the vertical axis according to the cut points suggested by Evenson et al. (2008).

#### Cognition

Testing of all cognitive and early academic learning measures was performed individually in a room without disturbances. A trained assessor was present and provided instructions, and, if the child preferred, a preschool staff member could join the child in the room, without influencing test procedures. Testing was divided into two blocks of three tests, with a (minimum) 15-min break after the first block, performed in a standardized order (Block 1: Early Years Toolbox (EYT) Go/No-Go, EYT Numbers, and Head-Toes-Knees-Shoulders (HTKS) task and Block 2: EYT Mr. Ant, EYT Card Sorting, and EYT Expressive Vocabulary). All tasks were translated into Norwegian. As some of the tasks lasted longer the better the children performed, the total test duration varied from 30 to 45 min. After completing the measurements, children received a sticker of physically active cartoon figures. Prior to the data collection, all assessors were thoroughly trained in the test procedures, including practice with children. The trained assessor also considered the validity of each task regarding children’s understanding of the task instructions.

##### Self-Regulation

Self-regulation was assessed by a structured, direct measure: The Head-Toes-Knees-Shoulders (HTKS) task. The HTKS assesses the ability of a child to integrate and apply executive function skills to control and direct actions despite instructions that generate contrary impulses. Children were asked to pay attention to and remember instructions, inhibit the automatic responses generated, and instead execute gross motor movements in line with the task goals (Cameron Ponitz et al., 2008). During three blocks of 10 trials each, children were instructed to perform the opposite of the dominant response, such as “touch your head” when instructed to “touch your toes.” Rules increased in complexity with each new block. A correct response (opposite of the instruction) was awarded 2 points and self-correction to the correct response (after commencing or completing the incorrect response) was awarded 1 point. If children got ≥ 4 points on a block, they moved on to the next block. All three blocks started with brief practice with feedback. Possible scores ranged from 0 to 60, such that a higher score indicated better self-regulation.

The HTKS has shown construct validity in measuring children’s behavioral self-regulation in European samples (Cameron Ponitz et al., 2009; von Suchodoletz et al., 2013). As this task involved scoring by a trained assessor, we performed an inter-rater reliability test based on video scoring of 18 children prior to the data collection, which showed an intra-class correlation of 1.00.

##### Executive Function

To measure the three fundamental constructs of executive function—inhibition, working memory, and cognitive flexibility (Miyake et al., 2000; Best, 2010; Diamond, 2013; Zelazo et al., 2016)—we used the iPad-based Early Years Toolbox (EYT), which has shown strong reliability, convergent validity with existing measures, and developmental sensitivity (Howard and Melhuish, 2017).

Inhibition was measured using the EYT Go/No-Go task, which asked children to catch the fish (Go trial, 80%) by tapping the screen when they saw a fish and to avoid the shark (No-Go trial, 20%) by not tapping the screen when they saw a shark. After instructions and a practice round, the task proceeded with 75 stimuli divided evenly into three 1-min test blocks, separated by a short break with repetition of instructions. The animated stimulus (fish or shark) was visible for 1.5 s and separated by a 1 s interstimulus interval. Responses (children tapping the screen) <0.3 s were removed, as this indicates a response that was unlikely to be initiated in response to the stimulus. The outcome measure of inhibition was an impulse control score (product of proportional Go and No-Go accuracy), representing the strength of the prepotent response in relation to children’s ability to inhibit this response. Possible scores ranged from 0 to 1, where a higher score indicated better inhibitory control.

Visual–spatial working memory was measured using the EYT Mr. Ant task, which asked the children to remember spatial locations of an increasing number of “sticker(s)” located on Mr. Ant’s body. After instructions and a practice round, the task proceeded with three trials (or two trials if correct on the first two) at level one (remembering the location of one sticker), with each subsequent level increasing in difficulty by adding one sticker. The task lasted until the children completed level eight or, in all cases in the current sample, until failure on all three trials at the current level. The outcome measure was a score calculated as one point for each passed level (at least two of the three trials on one level performed accurately) in a row, starting from the first, plus 1/3 of a point for all correct trials thereafter. Possible scores ranged from 0 to 8, where a higher score indicated better working memory.

Cognitive flexibility was measured using the EYT Card Sorting task, asking the children to sort cards of red and blue rabbits and boats by either color or shape, into two locations (identified by a blue rabbit and a red boat). As the card sorting rules alternate, the children must switch between rules. After instructions and a practice round, the task proceeded with three levels of six trials each. On level one, children were asked to sort cards by color and on level two by shape. At least 5 out of 6 correct trials on both levels were required to proceed to level three, where children were asked to sort cards by color if they were surrounded by a black border or by shape if there were no black border. The outcome was the accuracy (number of correct sorts) on levels two and three (when the switch trials began). If accuracy on level two was higher than on level one, values from level one replaced values from level two. Possible scores ranged from 0 to 12, where a higher score indicated better cognitive flexibility.

#### Early Academic Learning

Early academic learning was assessed as early expressive vocabulary and early mathematical skills, referred to as “vocabulary” and “numeracy,” respectively, hereafter. These were also measured using the EYT (Howard and Melhuish, 2017; Howard et al., 2021).

Vocabulary was measured using the EYT Expressive Vocabulary task, consisting of 54 items with increasing difficulty, asking the children to verbally produce a correct label for the depicted nouns and verbs. The task continued until the child completed all items or was automatically stopped because they answered six items in a row incorrectly. We only accepted words pronounced in Norwegian. Vocabulary was scored as the number of correct items. Possible scores ranged from 0 to 54, where a higher score indicated better vocabulary.

Numeracy was measured using the EYT Early Numeracy task, consisting of 79 items of increasing difficulty that pertain to numerical concepts, spatial and measurement concepts, counting subset, matching digits and quantities, number, ordinality, cardinality, subitizing, patterning, numerical word problems, and equations. The starting point varied from item 1, 11, and 21 for children aged 3, 4, and 5 years, respectively. If the child provided three consecutive incorrect answers to begin with (on item 11 or 21), the app returned to the prior starting point. After five consecutive incorrect responses, the task was automatically stopped. Numeracy was scored as number of trials correct across the entire task. Possible scores ranged from 0 to 79, where a higher score indicated better numeracy skills.

#### Anthropometry, Parental Education, and Sleep

Body mass was measured to the nearest 0.1 kg using an electronic scale (Seca 899, SECA GmbH, Hamburg, Germany) with children wearing light clothing. Height was measured to the nearest 0.1 cm using a transportable Seca 217 (SECA GmbH, Hamburg, Germany). Body mass index (BMI; kg·m^−2^) was calculated. Children were categorized as normal weight (including underweight), overweight, or obese according to the age- and sex-specific BMI cut points by Cole et al. (2000) for descriptive purposes. Parental education level (highest education of mother or father) and children’s sleep time were reported by parent(s) or guardian(s) by questionnaire. Sleep was included as a covariate in our analyses due to its important role for learning, memory, and cognitive processing (Deak and Stickgold, 2010).

### Ethics Statement

The procedures and methods used in the ACTNOW study are conform to the ethical guidelines defined by the World Medical Association’s Declaration of Helsinki and its subsequent revisions (WMA, 2013). The study was approved by the institutional ethics committee and the Norwegian Centre for Research Data (reference number 248220). ACTNOW is registered in clinicaltrials.gov 7 August 2019, with identifier NCT04048967.^1^

Prior to all testing, each child’s parent(s) or guardian(s) provided written informed consent. Children were informed about the study and testing procedures prior to and during measurements according to their level of understanding.

### Statistical Methods

Children’s characteristics are provided as frequencies, means, and standard deviations (SD). Differences between boys and girls, age groups (younger/older), and included and excluded children were tested using linear mixed models including random intercepts for preschools. PA data were adjusted for wear time. Bivariate associations between explanatory (using SED, LPA, MPA, VPA, MVPA, and TPA as proxies for the intensity spectrum) and outcome variables were determined using linear mixed models on variables adjusted for wear time (PA), sex, and age.

The multivariate PA intensity signatures associated with self-regulation, executive function, and early academic learning were determined using multivariate pattern analysis applied to the uniaxial and triaxial intensity spectra, equivalent to its previous application to accelerometer data (Aadland et al., 2018, 2021; Nilsen et al., 2020). Partial least squares (PLS) regression analyses (Wold et al., 1984) were used to determine the association patterns between self-regulation, executive function, and early academic learning (outcome variables) and both uniaxial (33 intensity variables from the vertical axis) and triaxial intensity spectra (99 intensity variables) included as explanatory variables in separate models. Briefly, PLS regression decomposes the explanatory variables into orthogonal linear combinations (PLS components), while simultaneously maximizing the covariance with the outcome variable. Thus, PLS regression is able to handle completely collinear variables through the use of latent variable modeling (Wold et al., 1984). Models were validated using Monte Carlo resampling (Kvalheim et al., 2018) with 1,000 repetitions by repeatedly and randomly keeping 50% of the subjects as an external validation set. For each model, we used target projection (Kvalheim and Karstang, 1989; Rajalahti and Kvalheim, 2011) followed by reporting of explained variance (*R*^2^) and multivariate correlation coefficients with 95% confidence intervals (CIs) to show the importance of each PA intensity variable in the multivariate space (Rajalahti et al., 2009a,b; Aadland et al., 2019a). To adjust for sources of variation and confounding, we obtained residuals from linear regression models using self-regulation, executive function, and early academic learning (model 1 adjusted for sex age, model 2 additionally adjusted for BMI, sleep, and parental education level) and PA variables (model 1 adjusted for sex, age, and wear time, model 2 additionally adjusted for BMI, sleep, and parental education level) as outcomes, prior to performing the multivariate pattern analysis. In secondary analyses, we determined association patterns separately in boys and girls and younger and older children (defined by median split). A value of *p* ≤0.05 was considered statistically significant. Multivariate pattern analyses were performed using the commercial software Sirius version 11.0 (Pattern Recognition Systems AS, Bergen, Norway), while all other analyses were performed using the SPSS software, version 28.0 (IBM SPSS Statistics for Windows, Armonk, NY: IBM Corp., United States).

## Results

### Children’s Characteristics

Characteristics of the 711 included children are shown in Table 1. Girls performed significantly better than boys on all tests of self-regulation and executive function (*p* < 0.002), whereas early academic learning outcomes were similar for boys and girls (*p* = 0.456 for vocabulary and *p* = 0.052 for numeracy). Boys had significantly higher TPA (cpm) and spent more time (min/day) in all PA intensities, including SED, than girls (*p* < 0.001). The older children (4.6–6.5 years old) performed significantly better than the younger children (2.7–4.6 years old) on all tests of self-regulation, executive function, and early academic learning (*p* < 0.001) and had higher TPA and spent more time in VPA, MVPA, and SED (*p* < 0.040) and had less time in LPA (*p* < 0.001), than the younger children. MPA did not differ among younger and older children (*p* = 0.421).

**Table 1:** Children’s characteristics.

	Total sample	Boys	Girls	Younger	Older
	*n* = 711	*n* = 369	*n* = 342	*n* = 355	*n* = 356
Age (years)	4.6 (0.8)	4.5 (0.8)	4.6 (0.8)	3.9 (0.5)	5.2 (0.3)
BMI (kg/m^2^)	16.2 (1.5)	16.2 (1.4)	16.2 (1.6)	16.3 (1.5)	16.1 (1.5)
Weight status (%)					
Normal	84.5	89.2	79.5	86.2	82.9
Overweight	12.8	8.9	17.0	11.8	13.8
Obese	2.7	1.9	3.5	2.0	3.4
Sleep (min/day)	657 (44)	657 (44)	658 (43)	668 (41)	646 (44)^*^
Parental education level (%)					
≤ Upper secondary school	23.2	23.3	23.1	21.4	25.0
University/college <4 years	29.1	30.1	28.1	30.7	27.5
University/college ≥4 years	47.7	46.6	48.8	47.9	47.5
Physical activity					
Wear time (min/day)	765 (69)	767 (70)	763 (67)	761 (67)	769 (70)
Total PA (cpm)	696 (153)	729 (151)	659 (147)^*^	676 (144)	715 (159)^*^
SED (min/day)	540 (64)	531 (63)	549 (64)^*^	534 (64)	545 (64)^*^
LPA (min/day)	149 (21)	155 (21)	143 (19)^*^	153 (21)	145 (21)^*^
MPA (min/day)	38 (7)	41 (8)	35 (6)^*^	38 (7)	38 (8)
VPA (min/day)	38 (10)	40 (11)	36 (10)^*^	36 (10)	40 (11)^*^
MVPA (min/day)	76 (17)	81 (17)	71 (15)^*^	74 (16)	79 (17)^*^
≥60 min MVPA/day (%)	85.4	90.8	79.5^*^	83.1	87.6
Self-regulation (score)	15.2 (18.0)	13.3 (16.9)	17.4 (18.8)^*^	6.8 (11.9)	23.7 (19.0)^*^
Executive function (score)					
Inhibition	0.60 (0.24)	0.56 (0.24)	0.65 (0.24)^*^	0.49 (0.23)	0.71 (0.19)^*^
Working memory	1.53 (0.97)	1.42 (0.99)	1.65 (0.93)^*^	1.11 (0.91)	1.95 (0.82)^*^
Cognitive flexibility	6.85 (3.05)	6.49 (3.03)	7.24 (3.01)^*^	5.95 (3.00)	7.74 (2.87)^*^
Early academic learning (score)					
Vocabulary	26.3 (9.6)	26.1 (9.6)	26.6 (9.5)	21.6 (8.5)	31.0 (8.2)^*^
Numeracy	31.5 (15.1)	30.5 (15.2)	32.0 (14.9)	22.5 (11.4)	40.4 (12.8)^*^

All values are mean (SD) if not otherwise stated. BMI, body mass index; SED, sedentary time; LPA, light physical activity; MPA, moderate physical activity; VPA, vigorous physical activity; and MVPA, moderate to vigorous physical activity. Weight status defined by the Cole et al. (2000) criteria. Physical activity level defined by the Evenson et al. (2008) cut points applied to the vertical axis. All data adjusted for physical activity valid days = ≥3 weekdays and 1 weekend day. Only subjects with valid data on all variables were included in analysis.

* Significant difference between boys and girls, and between younger and older children, *p* ≤ 0.05.

The included children performed significantly better on all measures of self-regulation, executive function, and early academic learning (*p* < 0.001) than the excluded children. The included children also had significantly higher TPA and spent more time in MPA, VPA, and MVPA (*p* < 0.001) and had less time in LPA (*p* = 0.017) than the excluded children. SED did not differ between the groups (*p* = 0.208).

### Association Patterns

Bivariate associations between (proxy) explanatory and outcome variables are shown in Table 2. We found significant negative associations between LPA and inhibition (*r* = −0.10) and between SED and numeracy (*r* = −0.08). Associations with numeracy were significant and positive for MPA, VPA, MVPA, and TPA (*r* = 0.08–0.10). Also, we found positive associations between self-regulation, executive function, and early academic learning (*r* = 0.21–0.32).

**Table 2:** Bivariate associations (standardized regression coefficient) among and between independent and dependent variables.

S. No.	Variables	1	2	3	4	5	6	7	8	9	10	11	12
1.	Sedentary time	–											
2.	Light PA	**−0.90**	–										
3.	Moderate PA	**−0.91**	**0.72**	–									
4.	Vigorous PA	**−0.73**	**0.38**	**0.73**	–								
5.	MVPA	**−0.86**	**0.56**	**0.90**	**0.95**	–							
6.	Total PA	**−0.80**	**0.51**	**0.77**	**0.96**	**0.94**	–						
7.	Inhibition	0.06	**−0.10**	−0.03	0.03	0.00	0.01	–					
8.	Working memory	−0.03	0.04	0.01	0.02	0.01	0.03	**0.27**	–				
9.	Cognitive flexibility	0.06	−0.05	−0.05	−0.05	−0.06	−0.07	**0.13**	**0.14**	–			
10.	Language	−0.02	0.01	0.02	0.04	0.04	0.04	**0.21**	**0.27**	**0.21**	–		
11.	Numeracy	**−0.08**	0.04	**0.10**	**0.09**	**0.10**	**0.08**	**0.28**	**0.32**	**0.26**	**0.55**	–	
12.	Self-regulation	−0.01	−0.01	0.02	0.04	0.03	0.04	**0.18**	**0.24**	**0.24**	**0.36**	**0.47**	–

PA variables (1–6) adjusted for sex, age, and wear time; cognitive and learning variables (7–12) adjusted for sex and age. PA, physical activity; MVPA, moderate to vigorous physical activity. Significant associations at *p* ≤ 0.05 are highlighted in boldface.

**Figure 2:**
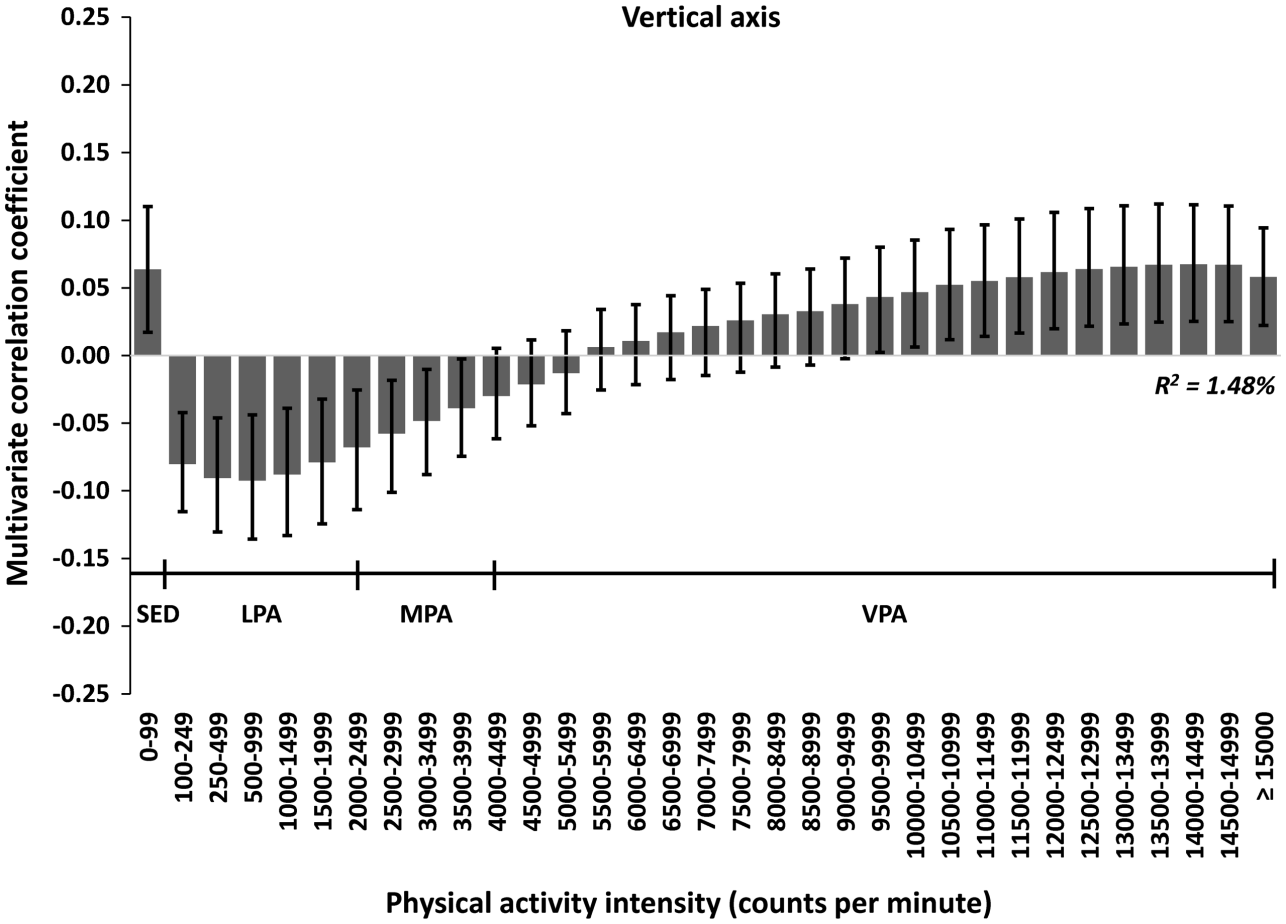
The multivariate physical activity signature for the uniaxial spectrum associated with inhibition in preschoolers. Results are reported as multivariate correlation coefficients. The model (PLS regression) is adjusted for sex, age, wear time (only PA variables), BMI, parental education level, and sleep (model 2).

**Figure 3:**
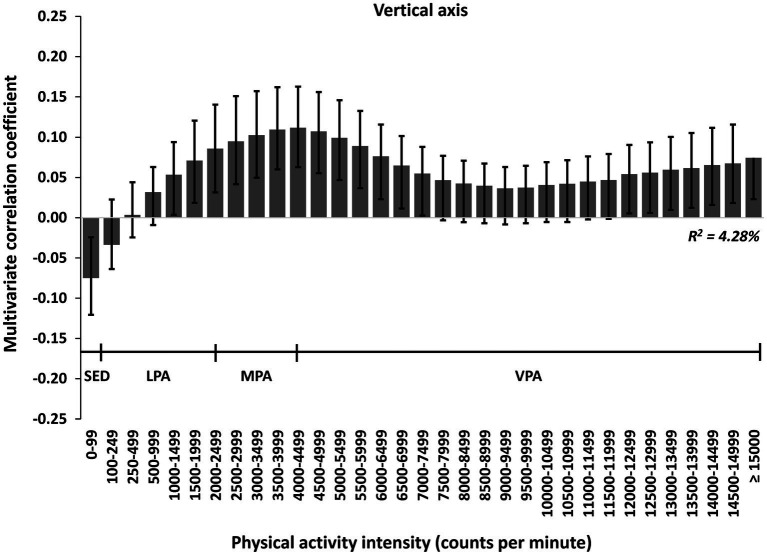
The multivariate physical activity signature for the uniaxial spectrum associated with numeracy in preschoolers. Results are reported as multivariate correlation coefficients. The model (PLS regression) is adjusted for sex, age, wear time (only PA variables), BMI, parental education level, and sleep (model 2).

In multivariate pattern analyses, we found a significant association between PA and inhibition (*R*^2^ = 1.48%, 2 PLS components; Figure 2), but no associations with working memory or cognitive flexibility, when using uniaxial data. The associations with inhibition were positive for time spent in 0–99 cpm and intensities ≥8,500 cpm, and negative for time spent in intensities between 100 and 3,499 cpm. Also, we found a significant association between PA and numeracy (*R*^2^ = 4.28%, 4 PLS components; Figure 3), but no association with vocabulary, when using uniaxial data. The associations with numeracy were positive for time spent in intensities between 1,000 and 7,499 cpm and ≥ 13,000 cpm, and negative for time spent in 0–99 cpm. We found no significant association between PA and self-regulation.

For triaxial spectra, we found a significant association between PA and numeracy (*R*^2^ = 1.12%, 1 PLS component, Supplementary Figure 1). No other associations were significant. As associations for the triaxial PA spectra were weaker than for the uniaxial spectrum, we used the uniaxial spectrum for the subgroup analyses.

In subgroup analyses, we found a significant association between PA and self-regulation in boys (*R*^2^ = 1.70%, 1 PLS component, Supplementary Figure 2), but not in girls, younger children, or older children. The associations with self-regulation in boys were positive for time spent in intensities ≥500 cpm, and negative for time spent in 0–99 cpm. Further, we found significant associations between PA and inhibition in girls (*R*^2^ = 3.12%, 2 PLS components) and older children (*R*^2^ = 3.33%, 2 PLS components; Supplementary Figures 3A,B), but not in boys and younger children. The associations with inhibition in girls were positive for time spent in 0–99 cpm and intensities ≥5,500 cpm, and negative for time spent in intensities between 100 and 2,499 cpm. The associations with inhibition in older children were positive for time spent in 0–99 cpm and time spent in intensities ≥10,000 cpm, and negative for time spent in intensities between 100 and 3,499 cpm. We also found significant associations between PA and numeracy in boys (*R*^2^ = 5.58%, 3 PLS components) and older children (*R*^2^ = 7.27%, 4 PLS components; Supplementary Figures 4A,B), but not in girls and younger children. The associations with numeracy in boys were positive for time spent in intensities ≥1,500 cpm, and negative for time spent in 0–99 cpm. The associations with numeracy in older children were positive for time spent in intensities between 1,500 and 7,499 cpm and ≥ 12,500 cpm, and negative for time spent in 0–99 cpm.

## Discussion

Using the novel approach of multivariate pattern analysis, we investigated association patterns for both uniaxial and triaxial PA intensity spectra with self-regulation, executive function, and early academic learning in 3–5-year-old children. For the triaxial PA spectra, we found a weak association pattern with numeracy, but no significant associations with other outcome variables. For the uniaxial PA spectrum, we found weak positive association patterns with numeracy and inhibition. Subgroup analyses for the uniaxial PA spectrum showed that the association between PA and numeracy only was evident in boys and older children, while the association between PA and inhibition only was evident in girls and older children. In addition, we found a weak positive association between PA and self-regulation in boys. We did not find any significant associations between PA and working memory, cognitive flexibility, or vocabulary.

Our finding of weak positive associations between moderate and vigorous PA intensities and numeracy are consistent with the findings of Bai et al. (2021) who found a positive association for both total PA and MVPA with cognitive school readiness (which also included colors, sizes, shape recognition, and comparison ability) in preschoolers. However, other studies in preschoolers have reported no associations between MVPA and numeracy-related outcomes (Becker et al., 2014; St. Laurent et al., 2018). Yet, since the assessment of outcomes differs with our study and across these studies math achievement by Becker et al. (2014), number recognition by St. Laurent et al. (2018), and school readiness by Bai et al. (2021), the findings may not be directly comparable. Also, Becker et al. (2014) only included assessment of PA during outdoor recess sessions, and thus excluded indoor activities and activities after preschool hours. Moreover, the previous studies (Becker et al., 2014; St. Laurent et al., 2018; Bai et al., 2021) had small sample sizes, used 15-s epoch length, and did not include the entire PA intensity spectrum in their analyses. Since including a high-resolution PA intensity spectrum can capture more information of movement behaviors of relevance for the outcomes than traditionally applied descriptors of PA (Aadland et al., 2019a, 2021; Nilsen et al., 2020), findings of Becker et al. (2014), St. Laurent et al. (2018) and Bai et al. (2021) are limited by relying on only a few gross intensity categories. Inclusion of the whole intensity spectrum has been called for in several previous studies (Poitras et al., 2016; van der Ploeg and Hillsdon, 2017).

Our findings showing an association between PA and numeracy but not between PA and vocabulary are consistent with findings from intervention studies in children and youth, showing beneficial effects of PA on math performance, while evidence regarding language-related outcomes are inconclusive (Donnelly et al., 2016; Singh et al., 2018). However, in line with the present study, previous observational studies examining associations between PA and academic achievement in school-aged children and youth show weak positive associations for some academic areas but no associations for others, though findings are inconsistent across studies (Rasberry et al., 2011; Donnelly et al., 2016). Limited evidence exists for associations with intensity-specific PA (Donnelly et al., 2016). Nonetheless, based on the present study findings, and the prevailing evidence on the relationships between PA and academic achievement irrespective of study design, PA appears to influence numeracy to a larger extent than vocabulary/literacy. With respect to intervention studies, such findings could derive from physically active lessons being more easily implemented with numbers, counting, and the use of mathematical concepts than with vocabulary/literacy (Donnelly et al., 2016; Singh et al., 2018). At the same time, vocabulary and literacy may have a greater focus and higher quality learning practices than numeracy-related practices in typical preschool settings where children are not necessarily physically active, possibly resulting in language learning being more ubiquitous than numeracy learning. However, information regarding PA content and context is needed to further address these hypotheses. We further speculate that the lack of inter-relationships between PA and vocabulary in the present study could relate to the lack of associations found between PA and both self-regulation and executive function since these cognitive functions play an essential role in learning processes (Barenberg et al., 2011; Donnelly et al., 2016; St. Laurent et al., 2018). However, bivariate associations in the present study showed that all constructs of self-regulation and executive function were correlated with both measures of early academic learning. Thus, there is no obvious reasons to expect that such inter-relationships could differently affect the associations for numeracy and vocabulary.

We found a weak association between PA and inhibition. However, it is difficult to interpret this association pattern showing that associations for both SED and VPA were positive, while associations for LPA and MPA were negative. A positive association between VPA and inhibition is different from findings by Cook et al. (2019), showing no associations for total PA or MVPA with inhibition. Furthermore, Cook et al. (2019) found no associations between PA and cognitive flexibility, but a negative association between PA and working memory, and suggested that free play does not enhance development of executive function. The positive association between SED and inhibition in the present study indicates that SED might facilitate inhibition in preschoolers and underlines the need to explore the influence of various sedentary behaviors. Findings of previous studies indicate that reading and/or being read to are associated with beneficial cognitive development while screen time, and in particular TV-viewing, is not or negatively associated with cognitive development in preschool-aged children (Carson et al., 2015). Importantly, the findings of Carson et al. (2015) are based on a small number of studies with low sample sizes. However, as the present study did not include information on types of sedentary behaviors, we can only speculate on the cognitive benefits of participating in activities typically characterized as sedentary, such as reading books/being read to, solving puzzles, playing with Lego, learning activities during group time or watching/using various screens. Therefore, future studies should include observation of PA and SED content and context to improve knowledge on associations between accelerometry data and executive function.

Even if individual variation in executive function development is present already in the preschool years (Liebermann et al., 2007; Garon et al., 2008), evidence suggests that brain structures that support executive function mature into adolescence and early adulthood (Tamnes et al., 2010). Therefore, we recognize that the process of executive function differentiation in children is slow and that particular tasks may be more or less sensitive to developmental improvements (Diamond, 2006; Lee et al., 2013). However, the present study found that girls and older children scored better on self-regulation and all three executive function components compared to boys and younger children, which may support that our assessments have captured individual differences in cognitive development. Also, our subgroup analyses revealed that PA was associated with inhibition among girls and older children only, which might be explained by differences in cognitive developmental status, supported by previous studies showing more consistent positive associations between PA and cognitive outcomes in school-aged children (Donnelly et al., 2016).

Previous studies have shown positive associations between PA and self-regulation (Becker et al., 2014; Bai et al., 2021) using the HTKS task, as also used herein. Because HTKS has an inherent motor component requiring children to perform bodily movements, our finding showing no association between PA and self-regulation was somewhat unexpected. However, this finding might be consistent with the weak or non-existent associations between PA and executive function. Indeed, HTKS is dependent on involvement of all executive functions in requiring children to inhibit prepotent cognitive responses and bodily movements by focusing and remembering specific rules (Cameron Ponitz et al., 2008, 2009). Also, as cognitively engaging and complex exercise is hypothesized to have a stronger impact on cognitive development than non-engaging and simpler exercises (Best, 2010; Diamond, 2015; Schmidt et al., 2015; Tomporowski et al., 2015), the lack of associations found between PA and both executive function and self-regulation in the present study might relate to insufficient cognitive demands embedded in PA. Notably, our subgroup analyses showed a weak positive association between PA and self-regulation among boys. We have no good explanation for a possible different association for boys and girls. Given the very weak association in boys, it could be a chance finding. We may also speculate whether poorer self-regulation (Gestsdottir et al., 2014) but higher PA levels in boys than in girls mean that boys inherently need more movement than girls and that this stimulus to a larger extent in boys than in girls positively affect self-regulation. This hypothesis is consistent with greater benefit of physically active learning in school-aged boys than in girls (Resaland et al., 2018).

Our finding of lower explained variances when using triaxial as compared to uniaxial PA intensity spectra, contrasts previous studies with children and preschoolers showing higher explained variances using triaxial data (Aadland et al., 2019b, 2021). The previous findings show that triaxial data can capture information about PA that uniaxial data cannot and that this information is relevant for the outcome. Nilsen et al. (2020), however, found that uniaxial and triaxial accelerometry provided rather similar information in relation to fundamental motor skills in preschoolers. A possible reason for our result is inclusion of many variables with little or no relevant information for the outcome (i.e., “noise”). This may alter the covariance structure and number of valid PLS components and thus lead to poorer model fit and weaker associations as expressed by the target projected component (Kvalheim, 1985).

### Strengths and Limitations

First, the main strength of this study was the inclusion of the whole PA intensity spectrum (based on a 1-s epoch length) from both uniaxial and triaxial accelerometry and the use of multivariate pattern analysis for determination of joint associations for the PA intensity variables with the outcomes. Second, the relatively large sample size facilitated generalizability of findings and allowed for us to explore sex- and age-specific associations. Third, we adjusted for sex, age, wear time, BMI, parental education level, and sleep. Further adjustment for preschool did not affect findings (results not shown). Therefore, we argue that the present study provides unique and nuanced evidence of association patterns between PA and key aspects of cognitive and learning outcomes in 3–5-year-old children.

Accelerometers are not without limitations and are not able to correctly capture certain activities that may characterize young children’s movement behaviors, such as rolling, crawling, climbing, cycling, and other non-load bearing activities. Moreover, accelerometers are not able to provide information on type or context of PA or sedentary behaviors. In addition, the cross-sectional design lacks a temporal relation between the exposure and outcome, meaning that we cannot demonstrate causal relationships. We recommend that future studies seek to determine longitudinal associations and pathways between PA and cognitive and learning outcomes in preschoolers. Finally, the present study did not investigate associations between PA and motor skills. Since motor skills have been shown to associate with cognitive and learning outcomes in children (Diamond, 2000; Cameron et al., 2012; Aadland et al., 2017), future studies should explore associations between PA, motor skills, and cognition.

## Conclusion

The purpose of the present study was to investigate associations between PA and cognitive and learning outcomes in children aged 3–5 years old. We included a large sample of preschoolers and explored PA association patterns by using the novel approach of multivariate pattern analyses. Our findings revealed weak association patterns between the PA intensity spectrum and numeracy and inhibition. Subgroup analyses showed stronger associations between PA and numeracy in boys and older children and stronger associations between PA and inhibition in girls and older children than in their peers, respectively. We recommend that future studies include observation of PA and SED content and context to help interpret associations with accelerometry data. Future studies should also determine longitudinal and causal relationships to further address the role of PA in promoting child cognitive development and learning.

## Data Availability Statement

The datasets presented in this article are not readily available because Privacy protection regulations restrict sharing of data. Requests to access the datasets should be directed to kbva@hvl.no.

## Ethics Statement

The studies involving human participants were reviewed and approved by The institutional ethics committee and the Norwegian Centre for Research Data. Written informed consent to participate in this study was provided by the participants’ legal guardian/next of kin.

## Author Contributions

EA, KA, and SH contributed to conception and design of the study. KV, EA, and KA organized the database and wrote sections of the manuscript. KV and EA performed the statistical analysis. All authors contributed to manuscript revision, read, and approved the submitted version.

## Funding

The study was funded by the Research Council of Norway (grant number 287903), the County Governor of Sogn og Fjordane, the Sparebanken Sogn og Fjordane Foundation, and the Western Norway University of Applied Sciences.

## Conflict of Interest

The authors declare that the research was conducted in the absence of any commercial or financial relationships that could be construed as a potential conflict of interest.

## Publisher’s Note

All claims expressed in this article are solely those of the authors and do not necessarily represent those of their affiliated organizations, or those of the publisher, the editors and the reviewers. Any product that may be evaluated in this article, or claim that may be made by its manufacturer, is not guaranteed or endorsed by the publisher.

## Footnotes

^1^ https://clinicaltrials.gov/ct2/show/NCT04048967?term=actnowandrank=1
