# Supplementary material for: The Multivariate Physical Activity Signatures Associated With Self-Regulation, Executive Function, and Early Academic Learning in 3–5-Year-Old Children
Source: Front Psychol. 2022 Apr 5;13:842271. doi: 10.3389/fpsyg.2022.842271 (PMC9037291; doi:10.3389/fpsyg.2022.842271)
Supplement: Supplementary file 1 [file Data_Sheet_1.PDF]

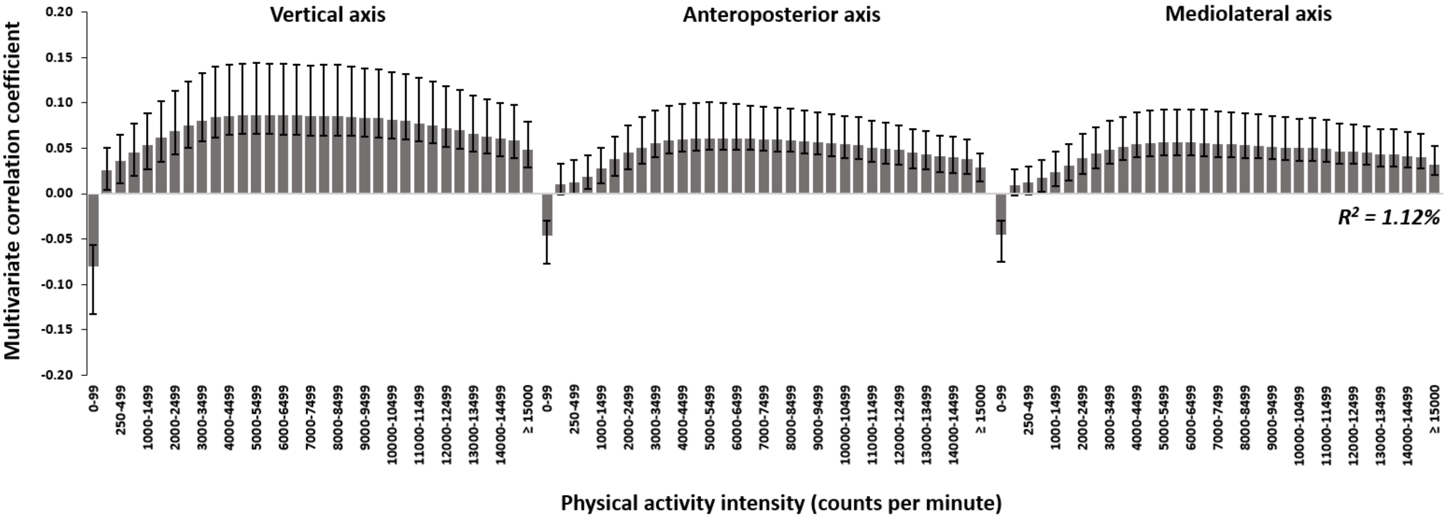

Figure 1. The multivariate physical activity signature for the triaxial spectrum associated with numeracy in preschoolers. Results are reported as multivariate correlation coefficients. The model (PLS regression) is adjusted for sex, age, wear time (only PA variables), BMI, parental education level, and sleep (model 2).

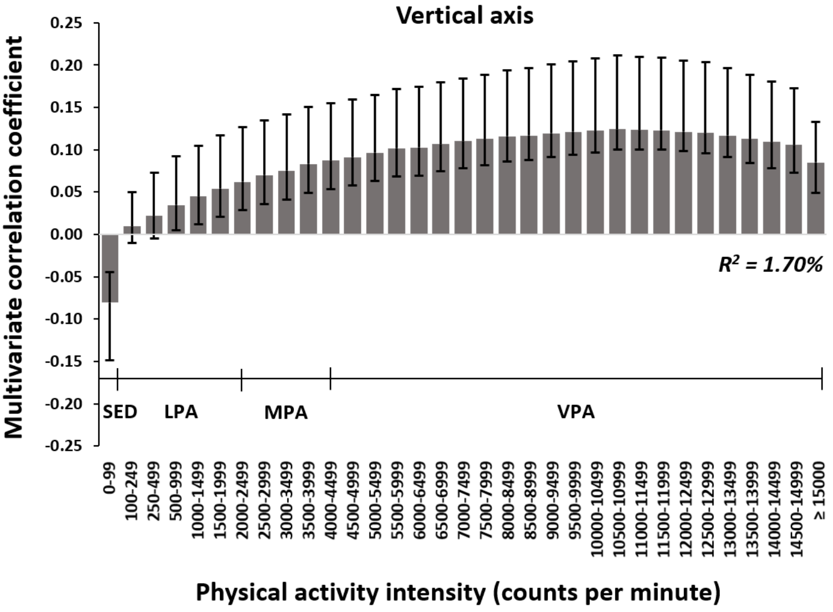

Figure 2. The multivariate physical activity signature for the uniaxial spectrum associated with self-regulation in preschool boys. Results are reported as multivariate correlation coefficients. The model (PLS regression) is adjusted for sex, age, wear time (only PA variables), BMI, parental education level, and sleep (model 2).

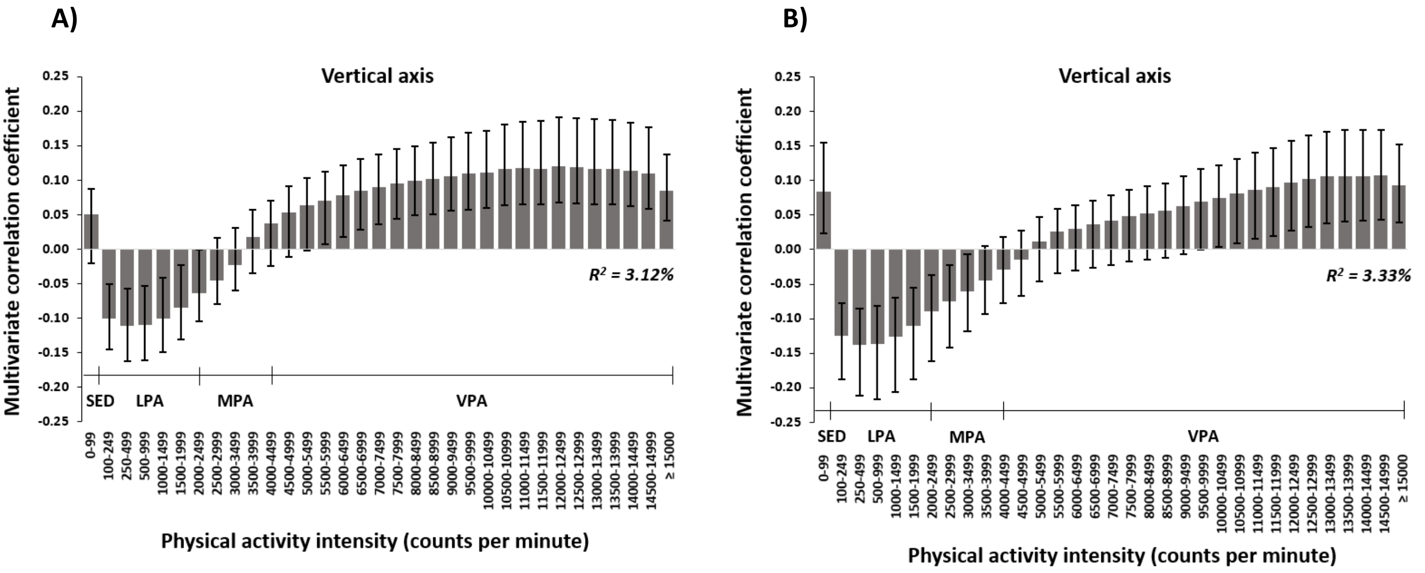

Figure 3 A-B. The multivariate physical activity signature for the uniaxial spectrum associated with inhibition in preschool girls **(A)** and older children **(B)**. Results are reported as multivariate correlation coefficients. The model (PLS regression) is adjusted for sex, age, wear time (only PA variables), BMI, parental education level, and sleep (model 2).

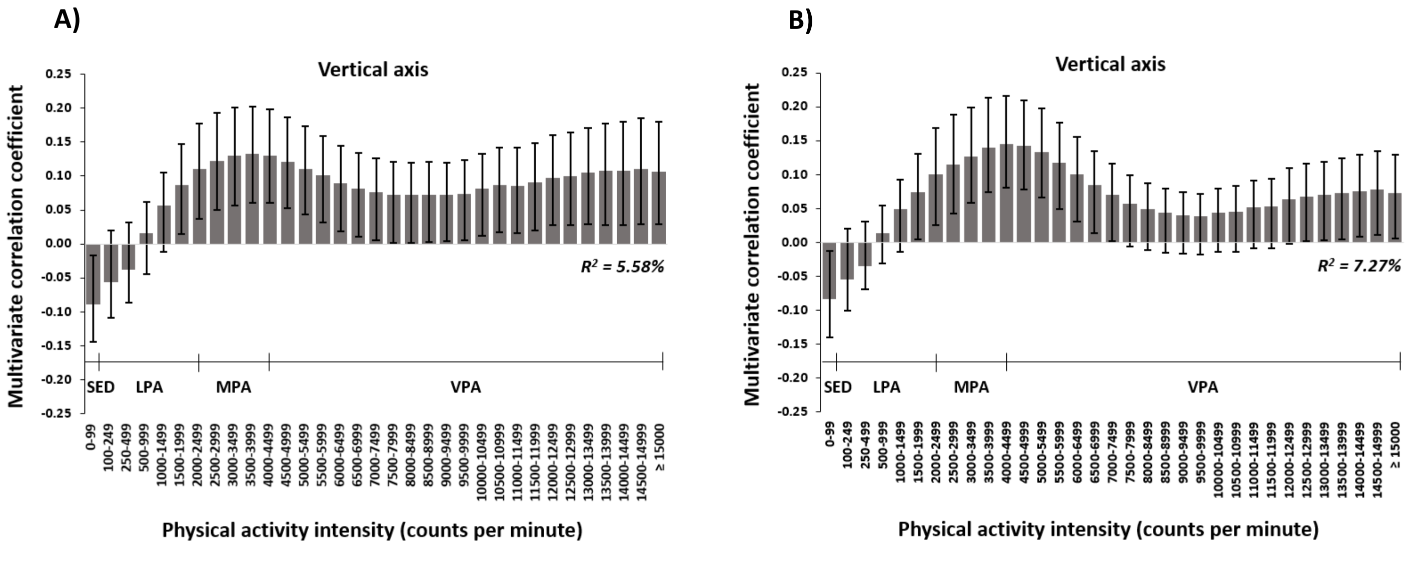

Figure 4 A-B. The multivariate physical activity signature for the uniaxial spectrum associated with numeracy in preschool boys **(A)** and older children **(B)**. Results are reported as multivariate correlation coefficients. The model (PLS regression) is adjusted for sex, age, wear time (only PA variables), BMI, parental education level, and sleep (model 2).
